# Co-morbid conditions in COVID-19 patients in Uttarakhand state of India

**DOI:** 10.7189/jogh.11.03029

**Published:** 2021-02-15

**Authors:** Divya Goel, Sudhir Kumar

**Affiliations:** Department of Biotechnology, Hemvati Nandan Bahuguna Garhwal University, Srinagar (Garhwal), Uttarakhand, India

India reported its first case of corona virus disease-19 (COVID-19) on 30 January 2020, a disease which by 2 November 2020 had infected 9 139 865 people and claimed the lives of 133 738 people nationwide (www.mohfw.gov.in). Almost all states are reeling under the effect of COVID-19, some experiencing more distress than others. A larger number of deaths had been attributed to co-morbid conditions all over the world and same is the case for India. Uttarakhand state lies in northern Himalayan region of India and has reported 71 256 confirmed COVID-19 cases and 1155 deaths till 22 November 2020 [1]. We analysed the data from reported cases of deaths in COVID-19 patients as recorded by the Uttarakhand state on its official website of Department of Medical Health and Family Welfare (www.health.uk.gov.in) till 2 October 2020 to find out the major co-morbid conditions for these patients. Uttarakhand reported 636 deaths by 2 October 2020, out of which cause of death was mentioned for 625 cases. As seen in COVID-19 related mortalities, in other countries, diabetes remained the major cause of co-morbidity followed by kidney related diseases, hypertension and cancer.

**Diabetes**: Diabetes has been identified as one of the leading cause for mortality in COVID-19 patients [2]. People with type 1 or type 2 diabetes have other associated medical conditions like hyperglycemia, weak immune system along with hypertension and cardiovascular diseases that makes them more vulnerable [3]. In India as well, diabetes continues to be leading co-morbid condition. Data analysis from the state of Uttarakhand till 2 October 2020 show that out of 636 reported deaths due to COVID-19, 65 people (10.2%) had either Type 1 or type 2 diabetes (www.health.uk.gov.in). Out of these 5 had developed diabetic ketoacidosis and 2 had diabetic nephropathy. Data suggests that both male and females with underlying diabetic condition and having average age above 60 are at heightened risk to COVID-19 related mortality. Centre for disease prevention and control (CDC) recommend continuing the medications and keep track of the blood glucose for people with diabetes [2].

**Figure:**
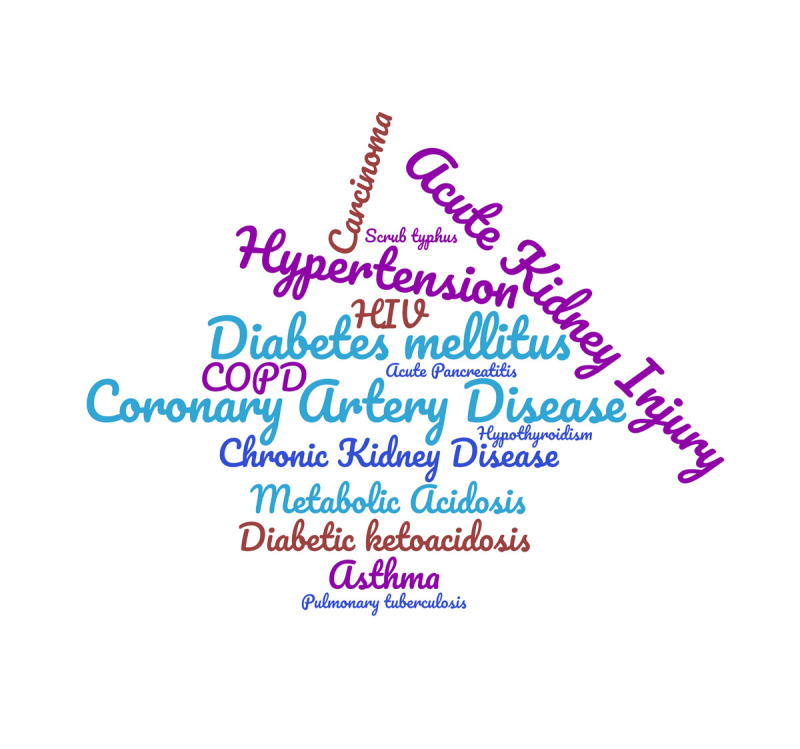
Photo: Major co-morbid conditions reported in deceased Covid-19 patients in Uttarakhand state till 2 October 2020. Font size is indicator of the number of times the word has been repeated in the data. Picture has been prepared in https://www.wordclouds.com/ by the author.

**Kidney disease**: Kidney disease like chronic kidney disease and acute kidney injury (AKI) are associated with the increased risk of the severe COVID-19 condition leading to mortality [2,4]. A study conducted in China reported a 27% incidence of AKI in 85 COVID-19-positive patients with an increased risk associated with people above the age of 60 years suffering from hypertension, and coronary artery disease [5]. We have found 22 deaths with chronic kidney disease as co-morbid condition in Uttarakhand state. A huge number of 62 deaths were reported as having acute kidney injury. Kidney diseases thus present a major challenge during the treatment of COVID-19.

**Hypertension**: People with hypertension have also been recognized as high risk group in COVID-19 infection [2]. In some reports, hypertension has been reported in as much as 30% of the COVID-19 cases [6]. Elderly people have many age related conditions and hypertension is one of them, making it a common co-morbid condition. In Uttarakhand, 26 instances were found where hypertension was mentioned in cause of death out of 636 COVID-19 related death cases (4.1%). Hypertension can also be the indication of other cardio vascular condition or diabetes, and thus increase the change of progression of COVID-19 infection to severe category.

**Cancer**: Cancer is generally considered a life threatening disease and the associated medical treatments like chemotherapy and radiotherapy tends to weaken the immune system of the body. A study done in China estimated that COVID-19 infected cancer patients are at 3.5 times larger risk of acquiring severe disease than other patients [7]. In Uttarakhand, we found that out of 636 reported deaths until 2 October 2020, 13 have cancer of varying stages. 6 had malignant form of the cancer with metastasis to other organs, while 4 have the lung carcinoma. Other type of cancers reported include 2 cases of oesophagal carcinoma, 2 cases of acute lymphoblastic leukemia, one case each of urinary bladder carcinoma and malignant mesothalioma.

**Cardiovascular conditions**: Pre-existing cardiovascular disease tends to increase the mortality rate in COVID-19 patients. COVID-19 infection can also cause problems in cardiovascular system like irregular heart beat (arrhythmia), myocardial injury, acute coronary syndrome and obstruction of a blood vessel by a blood clot (thromboembolism) [8]. We found 32 cases of coronary artery disease (CAD) among the 636 reported deaths in Uttarakhand. Several other cases listed cardiomyopathy as underlying cause.

**Other co-morbid conditions**: Conditions such as COPD (chronic obstructive pulmonary disease), HIV, asthma etc have been listed as co-morbid conditions that can aggravate the COVID-19 infection [2]. We found 2 cases of HIV, 2 of asthma and 3 with COPD among the expired patients.

**Age related mortality**: In Uttarakhand, first COVID-19 death was reported on 1 May 2020. Maximum deaths are reported in age groups of 51- 70 years contributing to 44% of total deaths from 1 May 2020 to 2 October 2020. Out of total deaths reported on 2 October 2020; 187 (30%) are female and 437 (70%) are male. Out of deceased 187 females, 74 (39.6%) were of age 50 years or less which is a worrying trend while only 30.6% deceased males were of same age group (**Figure 1**).

**Figure 1:**
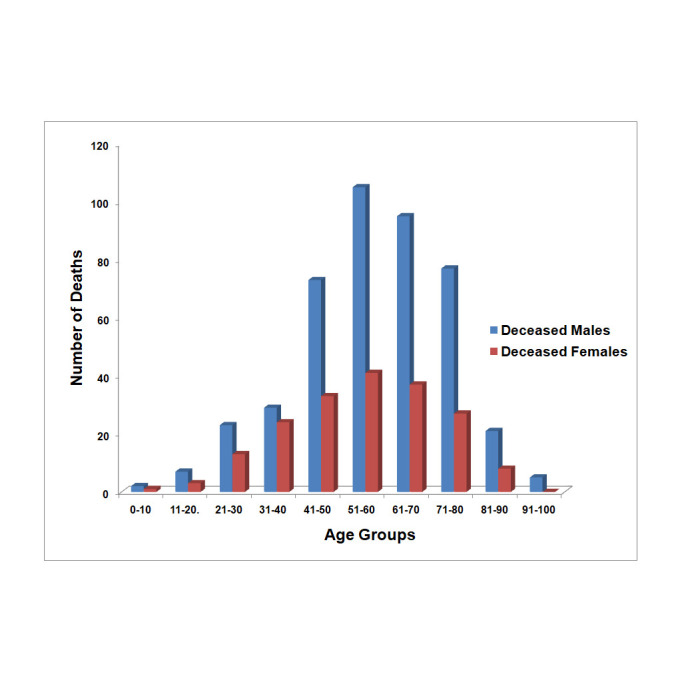
Comparative number of deaths among the males and females in Uttarakhand state in different age groups up to 2 October 2020 (www.health.uk.gov.in).

## CONCLUSION

Co-morbid conditions increase the risk of severe COVID-19 disease and also lead to increase in mortality among the patients. There is an urgent need to identify such conditions and their management. Due to COVID-19 pandemic, many of the patients already suffering from these diseases are being neglected while the diagnosis of new patients is also hampered severely. It is very important that these patients are diagnosed early and given adequate treatment. Another major worry for Uttarakhand state is the high rate of mortality among younger women (age <50 years). India has a large population and relatively less health facilities especially in states like Uttarakhand which has a challenging geographical area. Gradual increase in health facilities across state to ensure their reach to each and every person in the remotest of areas is the only way out of this pandemic.
